# Monthly Summary

**Published:** 1867-06

**Authors:** 


### MONTHLY SUMMARY.

---

*Liebig's Extract of Flesh.*—At the Pharmaceutical Conference, Nottingham meeting, last year, Messrs. Dean and Brady presented a paper containing the results of examination of this substance. Several samples were examined, but we are chiefly concerned with that prepared in South America, by Herr Giesbert, and sold with the approbation of Baron Liebig. In this, these gentlemen found a considerable quantity of gelatin.

Their investigations have not thrown much additional light on the subject. They found acid phosphate of potash, chloride of potassium, kreatin, a substance which they supposed to be kreatinine in combination with some acid, probably phosphoric, and a quantity of colloid bodies. They failed to find phosphate of magnesia which other observers have detected, but noticed during evaporation the evolution of ammonia which may have proceeded from the splitting up of the ammoniaco-magnesian phosphate. They consider that the value of the extract is greatest in proportion to the quantity of crystalloids present; and the better specimens, as judged by this test, deliquesce more readily than the others.

This paper has called out a note from Baron Liebig, who states that the manufactory at Fray Bentos in South America is superintended by one of his former assistants, that the extract is made according to his formula, that it is packed in tin canisters of 36 to 45 pounds each, and that none is sold until after analysis and approval by him. He denies that it contains any gelatin.

He says that the presence of this last named substance gives greater consistence to the extract, allows it to retain more water, and renders it more liable to mouldiness.. By excluding gelatin, the yield is diminished, so that only one pound of his extract is obtained from 34 pounds of fresh lean meat or 45 pounds of butcher's meat, including fat and bones. He objects to the tannin test for gelatin, as this reagent throws down from the cold water extract a precipitate having all the properties of tannate of gelatin and only to be distinguished from it by not gelatinizing when concentrated.

As for the difference in color and taste, he says that it is due to the difference in the sex and age of the animals yielding the meat for the extract. The flesh of oxen gives an extract of darker color and higher flavor, the latter resembling that of venison. Cow's meat furnishes an extract of lighter color and milder flavor. The flesh of animals under four years of age cannot be used for this purpose, as it yields a pappy flavorless extract. The extract of ox meat is richer in creatinine and sarkin than that of cow's meat. This extract is often adulterated with common salt. He mentions an extract made by Dr. Turner of Darmstadt which contains 26 per cent. more water than Liebig's extract besides 9 per cent. of salt.

Liebig states expressly that he has kept all the details of the process to himself, having given only general directions for the manufacture. He says that there have been but two special formulæ given, one in the Bavarian Pharmacopœia, the other in the Pharmacopœia Germanica, but he expressly declares that neither of these is his.

The question of the dietetic value of extracts of this kind has been raised at the Society of Arts, and Dr. Thudichum was called upon for his opinion. The doctor stated that they lacked the essential properties of nutriment. He considers that they contain a stimulant analogous to those belonging to tea and coffee, which acts strongly on the heart and brain. He says there is not so much nutriment in a teaspoonful of the extract as in a mouthful of meat. Beef tea contains ingredients, especially creatine, the action of which resembles that of theobromine, and also potassium salts, so useful in the production of muscular power. It has besides several acids such as lactic, which heightens the flavour of meat, and has much to do with the appetizing property of osmazome. Too strong a solution of extract of meat is as bad as too strong tea or coffee.

The nutritive elements of meat which are wanting in the extract were then reviewed. Albumen and syntonine are absent. The latter remains after albumen is dissolved out, and is insoluble in water, but soluble in dilute acids. Myochrome, the coloring matter of muscles resembles hæmatin and, like it, contains iron. It is precipitated along with albumen during boiling. There is not much gelatin in the extract. It contains however, like beef tea, inosite, which is a sort of sugar, animal dextrine, creatine, creatinine, lactic and inosic acids. In short Dr. Thudichum considers it to be a sort of beef-tea solidified, possessing the same merits and liable to the same objections, with somewhat less of nutritive matter, Of the whole solid contents of meat, not more than one-eighth or one-fifth are retained in beef-tea or Liebig's extract of meat.

---

*Narceine.*—Clinical observations of the effects of a constituent of opium, known as *narceine* would lead us to believe that in this latter we have an article which will cause tranquil sleep, followed by pleasant waking and freedom from headache and stomach derangement, and not cause constipation. M. Eulenburg considers narceine to be superior for its sedative effects to all other substances. It is particularly serviceable in surgical cases, and may be used either internally or externally. In the former case, the dose is from a sixth to a half a grain of the hydro-chlorate of narceine in solution.

---

*Pure Silver.*—At a recent meeting of the California Academy of Natural Science, Mr. Gutzkow presented a sheet of chemically pure silver, three feet in diameter, three ounces in weight, and as thin as fine paper. The color was beautifully white, and texture like fine lace. This sheet was made by mixing solutions of protosulphate of iron and sulphate of silver in a large dish, and the silver rose to the surface, and there formed into a sheet. Successive sheets will rise with each stripping. This easy mode of obtaining chemically pure silver is of much practical value.

---

*Catching Cold,* says Dr. Thomas Inman, is a common phrase for an attack of catarrh, but it is a very incorrect one. One year I suffered so very severely from a series of "colds" that my attention was drawn especially to them. I was then lecturer on Medicine, and nearly every night from five o'clock to six during the winter months, had to turn out from a warm room to go through all weathers, lecture for an hour in a theatre heated by a stove and lighted by gas, and then return again to my snuggery at home. When I felt a fresh cold beginning, I tried in vain to account for it, until I accidentally saw in Copland's dictionary that the most fertile cause of a cold was coming from a moist, cold air to a hot and dry room. This at once explained to me the reason of my frequent suffering, for I had invariably gone into my hot room straight from the cold. I of course soon changed my habit: dawdled in the hall while taking off my great coat, perambulated in rooms which had no fire in, went up and down stairs and the like, ere I went into my study, whose temperature was also reduced. Since then, I agree with a friend who says, "that a cold comes from catching hot;" and I am disposed to think that there is a strong analogy between a chilblain on a child's toes and a cold in a person's nose, throat, and lungs.—*Medical Mirror.*

---

*Probing Gun Shot Wounds.*—From Dr. V. Gelcich of Los Angels, Cal., we have received a communication relative to the above subject which is worthy of notice. He says that there is much difficulty in discriminating between bone and ball by the use of the ordinary probe. His probe is simply a piece of white pine wood, made in the shape of a probe, introduced into the wound, rubbed against the suspected object, and quickly withdrawn, when, if it has touched the ball, traces of lead will be found upon it. He says, by this simple instrument, while a medical officer in the United States Army, he saved the limbs of two men on whom amputation was about to be performed for gunshot wounds in the lower extremities; what was long supposed to be bone proving to be lead by the aid of the white pine probe.

A porcelain probe has been used to show the presence of lead in the same manner, but it is probable that a softer substance like wood is better. At least where the channel made by the ball is straight, or nearly so, and in many cases where a probe is not at hand, this, which could be extemporized from a bit of wood, would prove extremely valuable. In cases where the ball did not take a direct course, it seems as though a piece of pine wood might be secured to the metallic probe and do its office in a superior manner. Dr. Gelcich offers his discovery to the attention of surgeons.—*Sc. Amer.*

---

*Necrosis of the Lower jaw in Makers of Lucifer Matches.*—It has been comparatively only a few years since this peculiar disease has been known. Lorinser of Vienna first called attention to it in 1845. Numerous observations, however, have since been made, and the fact is as thoroughly established as any other.

It is remarkable that it is the lower jaw only which is first attacked, and indeed the upper jaw nearly always escapes, however prolonged the attack may be. So striking an exemption demands an explanation, and no theory of the origin of the disease can be accepted which does not account for this curious fact. Some have supposed that phosphorus has nothing to do with it; others that it is a sort of scrofula; others, again, that it is caused by the arsenic in the phosphorus; and still others that it is occasioned by the gas set free.

The probability is that the phosphoric vapour is absorbed and oxidized by the saliva, and thus applied to the bone, which it attacks and destroys. This view is corroborated by another remarkable fact. The disease does not readily occur while the teeth are sound. It would seem that decayed teeth allow the acid held in solution by the saliva to come in contact with the bone and to destroy it. This would account for the proneness of the poison to attack the lower jaw, since that is the point which would naturally be more liable to suffer, on account of the gravitation of the poisoned saliva towards the floor of the mouth.

---

*Trichiniasis.*—The scavenger habits of the rat certainly render the contents of his entrails living poison to the viler animal that devours them, and thus a prolific source of trichinæ in swine. A committee of the Vienna Medical Society have made an elaborate report in which they maintain that the disease also originates in the rat; a large percentage of rats examined in different towns and countries having been found trichinized. It is also found that the germs of trichinæ may be conveyed from infected meat to other food by the larvæ of flies; which shows how a rat or other animal may become trichinized without eating either trichinized flesh or intestines containing germs. Prof. Brown, in a lecture before the Society for the advancement of Science and Art, in this city, stated that this parasite originates almost entirely in the swine, and is there invisible to the naked eye. When flesh containing the trichinæ is introduced into the human stomach, the flesh is dissolved and the parasite unloosed from its cell. When this occurs the parasite is about one-thirteenth of an inch in length. Birth is then given to trichinæ, which straightway proceed to penetrate the whole muscular and flesh system through the alimentary canal. These young trichinæ are at first only 1,540 of an inch in length, and resemble a worm in spiral coil. By the time they traverse the system, however, they increase in size many fold, and then begin to make felt that terrible disease to which they have given that name. As first introduced into the animal they cause trouble only by the production of their offspring. The disease is first made apparent by pains in the joints, the head, and the spine, and the patient gradually wastes away and dies. The trichinæ do not create disease by eating away the flesh—which they are not fitted to do—but by hindering or closing up the forces and processes by which health is preserved. From one of the limbs of a girl who had who had died in this manner lately in Springfield, Mass., a portion of muscle was detached and subjected to microscopic examination. A square inch of this disclosed from 30,000 to 80,000. trichinæ.

